# Lactoferrin: Balancing Ups and Downs of Inflammation Due to Microbial Infections

**DOI:** 10.3390/ijms18030501

**Published:** 2017-03-01

**Authors:** Maria Elisa Drago-Serrano, Rafael Campos-Rodríguez, Julio César Carrero, Mireya de la Garza

**Affiliations:** 1 Departamento de Sistemas Biológicos, Universidad Autónoma Metropolitana Unidad Xochimilco (UAM-X), CdMx 04960, Mexico; dragome@yahoo.com; 2 Sección de Estudios de Posgrado e Investigación, Escuela Superior de Medicina, Instituto Politécnico Nacional (ESM-IPN), CdMx 11340, Mexico; citlicampos@gmail.com; 3 Departamento de Inmunología, Instituto de Investigaciones Biomédicas, Universidad Nacional Autónoma de México (IIB-UNAM), CdMx 70228, Mexico; 4 Departamento de Biología Celular, Centro de Investigación y Estudios Avanzados del Instituto Politécnico Nacional (CINVESTAV-IPN), CdMx 07360, Mexico

**Keywords:** lactoferrin, innate immunity, inflammation, infections

## Abstract

Lactoferrin (Lf) is a glycoprotein of the primary innate immune-defense system of mammals present in milk and other mucosal secretions. This protein of the transferrin family has broad antimicrobial properties by depriving pathogens from iron, or disrupting their plasma membranes through its highly cationic charge. Noteworthy, Lf also exhibits immunomodulatory activities performing up- and down-regulation of innate and adaptive immune cells, contributing to the homeostasis in mucosal surfaces exposed to myriad of microbial agents, such as the gastrointestinal and respiratory tracts. Although the inflammatory process is essential for the control of invasive infectious agents, the development of an exacerbated or chronic inflammation results in tissue damage with life-threatening consequences. In this review, we highlight recent findings in in vitro and in vivo models of the gut, lung, oral cavity, mammary gland, and liver infections that provide experimental evidence supporting the therapeutic role of human and bovine Lf in promoting some parameters of inflammation and protecting against the deleterious effects of bacterial, viral, fungal and protozoan-associated inflammation. Thus, this new knowledge of Lf immunomodulation paves the way to more effective design of treatments that include native or synthetic Lf derivatives, which may be useful to reduce immune-mediated tissue damage in infectious diseases.

## 1. Introduction

Lactoferrin (Lf) is a conserved iron-binding mammalian glycoprotein with antimicrobial activity, present in secretions that recover mucosal sites regarded as portals of entry and/or invasion of pathogenic agents [1]. Antimicrobial activity has been mostly characterized in Lf of bovine and human origin isolated from milk [2,3]. Mechanisms underlying the antimicrobial action of Lf result from both direct (microbiostatic and/or microbicidal) and indirect (immunomodulatory) effects [3,4,5]. At present, the therapeutic and prophylactic treatments for microbial infections that ameliorate both the antibiotic multiresistance and the inflammatory response have prompted the searching of agents that display both antimicrobial and modulatory properties such as Lf. This review is focused on the modulatory impact of Lf on the inflammatory response induced by infectious microorganisms, mainly in the gastrointestinal and respiratory tracts. The study of modulatory properties of Lf on inflammatory response has impacted in the biotechnological development of nanoparticle Lf formulations of potential clinical implications [6].

## 2. Lactoferrin: Distribution, Structure and Biological Function as an Antimicrobial

**Figure 1:**
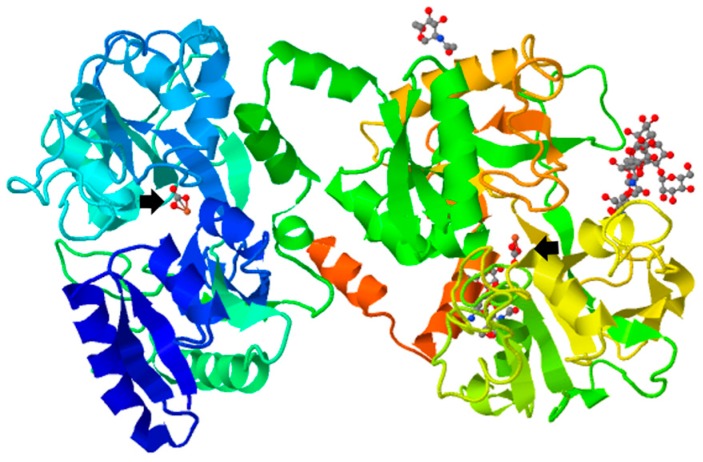
Tertiary structure of bovine ferric lactoferrin. Protein Data Bank (http://www.rcsb.org/pdb/explore.do? Structure Id = 1BLF). The bovine lactoferrin is represented in rainbow ribbon diagram showing two-lobe, four-domain polypeptide. Arrows show ferric ions.

Lf was isolated almost at the same time from human (hLf) [7] and bovine (bLf) [8] milk in 1960. Both glycoproteins are monomeric, with an approximated molecular weight of 80 kDa, and are highly cationic (pI 8.5–9). Tertiary structure (Figure 1) of Lf consists of two main N and C lobes organized in N1:N2 and C1:C2 domains. Both lobes are linked at N1 and C1 domains by a three-turn α-chain [9,10,11]. Each cleft lying between N1:N2 and C1:C2 domains can bind one ferric ion (Fe^3+^) (*K*_d_ = 10^−23^ M). In total, Lf can bind two ferric ions derived from the diet or from iron-loaded transferrin (holo-Tf), each one associated with a synergistically bound carbonate ion (CO_3_^2−^), and with the two domains of each lobe fully closed over the bound metal ion [9,12,13].

Lf was initially named lactotransferrin, due to being a milk glycoprotein that chelates iron. This protein belongs to the transferrin family, which includes the avian egg ovotransferrin (ovoTf) and the mammalian serum and lymph transferrin (Tf), but differs from other members of the family in its higher affinity for iron. Lf is synthesized by the mammary gland and then it is abundant in colostrum and milk, through which it has been suggested to participate in the initial protection in newborns [14,15,16,17,18,19]. Regarding the content, human mature milk is highly enriched in Lf (2.6 mg/mL) in comparison with bovine milk (0.09 mg/mL) [19,20,21]. The amino acid sequences of both proteins exhibits approximately 70% identity [22,23]. Lf is also present in many fluids and exocrine secretions, such as tears, saliva, and mucosal surfaces of the respiratory, urinary-reproductive and intestinal tracts; in these sites, Lf contributes to the primary innate-immune defense system of mammals that exerts antimicrobial activity against an extensive variety of pathogens [1,18,24,25,26,27,28].

Lf is also synthesized during the natural cellular development of promyelocytes to myelocytes, and was early recognized as an important component of the secondary granules of polymorphonuclear (PMN) neutrophils [29,30]. These cells store Lf (3–15 μg/10^6^ neutrophils) and release it at the sites of infection, which are acidic due to the activity of pathogens [17,18,31,32]. In plasma, Lf derives from neutrophils and its concentration is very low (0.4–2 μg/mL) [31]; nevertheless, in patients with sepsis the degranulation of activated neutrophils leads to secretion of significant levels of Lf (~0.2 mg/mL) into the bloodstream [9]. Neutrophils also release Lf in feces whose concentrations markedly increase during inflammatory processes such as inflammatory bowel disease (IBD), ulcerative colitis and Crohn’s disease, due to the response against pathogenic bacteria [33].

Physiologically, Lf can be found as a fully iron-loaded (holo-Lf) or iron-free protein (apo-Lf) [11,34,35,36]. The holo-Lf is conformationally more rigid and is more resistant to denaturation and proteolysis than the apo-Lf, but instead, apo-Lf is generally more effective against bacteria than holo-Lf [27,37,38,39,40]. In this regard, it has been reported that holo-Lf can be utilized as an iron source by several groups of microorganisms [39,41,42,43]. However, this is not always the case because studies on intestinal epithelial-barrier function and mucosal inflammation carried out in a Caco-2 cells model and macrophages activated with lipopolysaccharide (LPS) showed that both Lf forms effectively inhibited the pro-inflammatory response. Nevertheless, apo-Lf was more effective in downregulating inflammation, probably due to its ability to bind and neutralize LPS, as well as to neutralize microbial-derived antigens, thereby potentially reducing their pro-inflammatory effect [44].

Much evidence exists of the successful experimental use of Lf from different origins (human, bovine, porcine, caprine, camelid, and buffalo) against the growth of diverse pathogens. As mentioned above, most results indicate that Lf from different origins can exert bacteriostatic effects due to its iron-chelating activity, but it can also be bactericidal due to its interaction with LPS and porins in Gram-negative bacteria, or with teichoic acids in Gram-positive bacteria. These interactions lead to membrane damage and bacterial death [38,45,46,47,48,49,50,51,52,53,54]. Moreover, the antimicrobial activity of Lf is also highly dependent on its cationic properties, because the addition of positive charges to Lf via amidation enhances its antibacterial and antiviral properties and, in contrast, the addition of negative charges by acylation abolishes them [55].

As mentioned, Lf displays antiviral properties against common virus infections. These antiviral properties are related to its ability to block the cellular attachment or replication of virus by inducing type I interferons (α/β) with antiviral action [56]. Thus, Lf from diverse mammals shows a potent activity against replication of Human Immunodeficiency Virus, Cytomegalovirus, and Hepatitis C virus [57,58]. Less information exists about the microbicidal action of Lf against fungi and protozoa [59,60,61,62]. Very important is the finding that Lf synergizes with antibiotics and drugs, and even with other proteins of the innate immune system such as lysozyme and natural secretory IgA (sIgA) antibodies, potentiating the antimicrobial effect [63,64,65,66,67,68,69].

The largest external source of Lf is milk consumption. When consumed, Lf can be enzymatically cleaved by pepsin in the stomach and by trypsin in the small intestine. In adults, whereas hLf is completely degraded, about 60% of bLf resists proteolytic digestion mediated by pepsin [70]. Noteworthy to mention is that digestive tract of babies and infants has a relatively high pH and secretes low levels of pepsin, which allows for the innocuous transit of hLf and bLf into epithelial cells, which can be extremely important at this stage of life [71,72]. Nevertheless, native hLf derived from pancreatic juices and neutrophils is discharged into exocrine secretions of mucosal surfaces in adults, and thus it acts protecting those sites from invaders. In addition, diverse biological activities of Lf including the facilitation of iron absorption, modulation of mucosal immunity and stimulation of mucosal differentiation result of its interaction with Lf receptors (LfRs) expressed in the gastrointestinal cells [72,73]. One reason why Lf can be used as a pharmaceutical is because its activity is maintained in some of its component peptides after being cleaved by proteolytic enzymes, e.g., the peptides derived from the N1 terminus of Lf by pepsin, so-called lactoferricins (Lfcins), lack the iron-chelating activity, and are characterized by their strong cationic charge. Remarkably, Lfcins often show a higher microbicidal activity than the parental Lf as well as synergistically act with drugs and antibiotics against microbes [61,73,74]. Moreover, several Lfcins have been synthesized and experimented against microbes [75]. On the other hand, synthetic LFcin17–30, lactoferrampin (LFampin265–284), and a fusion peptide of both called LFchimera, have been successfully assayed against multiresistant bacteria, and also against bacteria that typically form biofilms [76,77]. This synthetic LFchimera has also been shown to be effective against parasitic protozoa [78,79,80]. Another synthetic LFchimera prepared by the fusion between Lfcin17–30 and Lfampin268–284, was effective against *Pseudomonas aeruginosa* by down-regulating pyocyanin, elastase and biofilm formation [81].

The presence of Lf in secretions and its various mechanisms of action allow this glycoprotein to combat all types of microbes that colonize mucosae in the different bodily regions. However, depending on the site, microbes can be exposed to different concentrations of Lf, to complexes of Lf with other proteins, or to diverse levels of Lf derivatives [82]. At the same time, Lf can help against the inflammatory process produced by strong immune reaction in infections. Therefore, all findings on Lf activities suggest that Lf and Lfcins can be of potential use as antimicrobial and anti-inflammatory compounds, either alone or as adjuncts to conventional antibiotics and drugs. In this sense, Lf is one of the most studied proteins since the commercial point of view, being highly appreciated as a nutraceutical in some countries, promoted as a supplement in diarrheic diseases, cancer, increasing immunity, improvement of memory, and several other conditions. Human Lf has been cloned in different vectors and expressed as recombinant (r-hLf) overall in eukaryotic systems which can glycosylate it, such as yeasts and fungi [83,84]; however, the best product is obtained from transgenic cows and plants [85,86,87]. Interestingly, r-hLf expressed in the cow mammary gland, enhanced systematic and intestinal immune responses in piglets used as a model of infants [88]. In addition, when the meat from the progeny of hLf transgenic cows was analyzed, no abnormalities of its nutrient composition were found [89]. Thus, the wide use of Lf in human health care is promissory. Next, we will review the effects of Lf as an anti-inflammatory protein in a number of infectious diseases in which it has been studied, mainly of gastrointestinal and respiratory tracts.

## 3. Lactoferrin as Anti-Inflammatory in Infectious Diseases

Inflammatory response is elicited by germ-line encoded pattern-recognition receptors (PRRs) expressed in many cell types that interact with their ligands from exogenous or endogenous origin, namely pathogen-associated molecular patterns (PAMPs), or danger-associated molecular patterns (DAMPs), respectively. Some PRRs comprise a large family of receptors such as Toll-like receptors (TLRs) [90,91,92]. Upon ligand binding, TLRs lead to signaling pathways resulting in the activation and translocation of the nuclear factor (NF)-κB to the nucleus. NF-κB modulates the expression of pro-inflammatory cytokines such as interleukin (IL)-1, IL-18, type-I interferon (IFN-α, and IFN-β), tumor necrosis factor (TNF) α, as well as chemoattractant cytokines (chemokines). Another class of PRRs includes Nod-like receptors (NLRs), some of which, such as NLRP1, NLRP3 and NLRP6, function as sensors or adaptors forming the “inflammasomes” [90]. Activation of inflammasomes by PAMPs and/or DAMPs induces signal pathways resulting in the activation of caspase-1 that cleaves the inactive pro-forms of cytokines (IL-1, and IL-18) to generate their active forms. Besides to generate active pro-inflammatory cytokines, some inflammasomes regulate cell death in response to microbial and endogenous danger signals [90,91,92,93].

Although Lf displays direct microbiostatic and/or microbicidal activities, indirect antimicrobial mechanisms have also been ascribed to its capability of modulating a wide array of humoral and cellular components of the innate and adaptive immunity [3,94]. Immunomodulatory role of Lf is due, in part, to its interactions with cell surface receptors that favor either elicitation of signal pathways, or Lf translocation into nucleus and gene targeting [95,96,97]. A summary of the modulatory effects of Lf on inflammation due to microbial infections is shown in Table 1.

### 3.1. Gastrointestinal Tract

#### 3.1.1. Gastrointestinal Tract Inflammation: An Overview

Throughout the gastrointestinal tract, Lf is present as an iron-binding multifunctional glycoprotein regarded as a natural compound able to inhibit the pathogens growth. Lf is also able to up- and down-modulate both humoral and cellular components of immunity involved in the regulation of the inflammatory response having a key role in maintaining gut homeostasis [3]. Balance of homeostasis results from the tight regulation of several events, since too little inflammation disrupts the process of tissue repairing and remodeling, whereas too much inflammation entails collateral impact by causing tissue damage with life-threatening consequences [128]. Mucosal compartment of the small intestine is a scenario where takes place a physiologic inflammatory response orchestrated by innate and adaptive mechanisms mediated by intestinal epithelial cells and by a wide array of immunocompetent cells at *lamina propria*, such as dendritic cells, macrophages and Tγδ lymphocytes, all with a key role in maintaining the gut homeostasis and combating infections [90]. However, in some infectious clinical conditions of the large intestine, such as IBD, inflammation has a double-edged sword role by either enabling or inhibiting cancer development and progression [90,128].

#### 3.1.2. Modulatory Effects of Lactoferrin on the Inflammatory Response Associated to Gut Infections

As it was commented before, antimicrobial activity of bLf against a wide array of pathogens has been profusely evidenced. In contrast, data about the regulatory role of bLf on the parameters of gut inflammation caused by enteropathogenic microorganisms have been provided by a limited number of articles. Findings from in vitro and in vivo models of infection show that bLf displays up- and down-modulatory effects on pro-inflammatory Th1 cytokine profile. The role of bLf on the resolution of infections by modulating mediators of inflammation has been documented in models of infection caused by several strains of enteropathogenic bacteria [98,99,100,101,102], and parasites [103]. Additionally, it has been found that Lf promotes the development of *Bifidobacterium*, one of the major genera of bacteria of the colon flora used as probiotics, in a manner independent of the iron saturation level of Lf [129,130]. This effect is believed to help maintaining the gut homeostasis.

Regarding to the gastric inflammation, the treatment with bLf or hLf as single agents or in combination with antimicrobial drugs, was found to favor eradication of bacteria and to protect against gastritis caused by *Helicobacter* *pylori* or *Helicobacter felis*, as described in murine models [104,131]. However, other human and murine trials did not support this finding, and even more, bacterial growth and gastric inflammation seemed to be enhanced by bLf or hLf administration [132,133]. These controversial data may reflect experimental or clinical settings of Lf treatment. Interestingly, Lf as a single component failed to eradicate the *H.* *pylori* infection but in combination with triple esomeprazole, clarithromycin and amoxicillin therapy and with probiotics, favored the resolution of infection and ameliorated the inflammatory response more effectively than in combination with two-antibiotic treatment, as described in experimental mice treated with r-hLf from transgenic goats, and also in trails of patients treated with native bLf. Interestingly, r-hLf not only inhibited the growth of *H. pylori*, but also suppressed the expression of two of its major virulence factors [133,134].

On the other hand, several studies have demonstrated the effect of Lf on modulating inflammation in the small intestine. For example, the anti-inflammatory activities of both r-hLf and native bLf have been tested in models of bacterial infection by *Shigella* *flexneri* in rabbits [98], and *Salmonella enterica* serovar Typhimurium in susceptible BALB/c mice [99]. Unlike with Lf-untreated animals, macroscopic and microscopic observations evidenced that both Lf treatments favored the resolution of infection and protected mice from tissue damage caused by the intestinal inflammation [98,99]. Mechanisms accounting for the anti-inflammatory role of bLf may result, in part, from the elicitation of sIgA response with a key role in luminal clearance of pathogens and in down-modulation of intestinal inflammation [108,116,117,135,136].

Although Lf may modulate inflammation by inhibiting the growth of pathogens through the iron chelating ability of apo-Lf, the iron-free form of Lf used in most studies, assays based on the murine typhoid model showed that pharmaceutical formulation of iron-saturated bLf (holo-bLf) enclosed in nanocapsules displayed both antimicrobial activity and modulatory properties on the inflammation. The latter was evidenced by up- and down-modulation of cytokines involved in innate and adaptive immune responses as well on hematopoietic cytokines, with a key role in the generation of both granulocyte (PMN neutrophils) or agranulocyte (monocytes/macrophages) phagocytes [6]. Thus, iron-loaded bLf nanocapsules seem to evoke the convergence of innate and adaptive immune responses of pro- and anti-inflammatory cytokines resulting in the protection toward typhoid infection and concomitant intestinal inflammation.

Down-modulatory effects of bLf on pro-inflammatory cytokines has also been documented in cultures of Caco-2 monolayer cells infected with recombinant *Escherichia* *coli* invasive strain harboring *inv* gene from *Yersinia* *pestis*; this strain is able to accomplish invasion but not intracellular multiplication within epithelial cells [100]. In this model, apo-bLf as well as holo-bLf decreased levels of IL-8 elicited by non-invasive *E. coli* wild type strain, and IL-8, IL-6 and TNFα by *E. coli* invasive. In addition, both apo- and holo-bLf inhibited an IL-8 increased response caused by *E. coli* invasive strain, but levels of this cytokine remained elevated. These findings suggested that the effect of bLf toward inflammatory mediators was iron-independent and that constant high IL-8 levels provided protection by inducing recruitment of phagocytes to combat the infection [100].

In the large intestine, the regulatory impact of bLf toward inflammatory response has been described in in vitro models of infection by adherent invasive *E. coli* (AIEC) strains with a presumable role in the pathogenesis of Crohn’s disease. This disease, along with ulcerative colitis, are two clinical entities of IBD characterized by an abnormal response to commensal bacteria colonizing the intestinal lumen [101,102]. AIEC LF82 strain is found in lesions of inflamed colon tissue in children suffering Crohn’s disease with a preponderant Th1 pro-inflammatory response [137]. Findings in the model of infection by AIEC LF82 indicated that bLf inhibited the bacterial invasion and the pro-inflammatory cytokine response of TNFα, IL-6 and IL-8 in epithelial monolayers and in cultures from colonic biopsies from patients with Crohn’s disease, suggesting a potential therapeutic role for bLf as antibacterial and anti-inflammatory agent [104]. Up-modulatory effects of bLf on inflammatory cytokines in response to bacterial infections have been found in assays of infection with AIEC LF82 in Caco-2 monolayers stimulated with IFN-γ to mimic the preponderant response of Th1 associated cytokines found in Crohn’s disease patients [105]. Data from these assays showed that in unstimulated infected cells, bLf inhibited both bacterial invasion and survival, while in infected cells primed with IFN-γ, bLf increased IL-8 production whereas counteracted the inhibitory effect of AIEC infection on ferroportin protein expression. Ferroportin is an iron exporter protein regulated by the inflammation that determines the survival of intracellular pathogens by reducing the intracellular iron levels. Apparent conflicting data described in the infection model with AIEC LF82 strain regarding the anti-inflammatory [104] versus pro-inflammatory [105] role of bLf seem to evidence two sides of the same coin, i.e., the ability of bLf to up- and down-modulate the inflammatory response and iron availability resulting in the resolution of infection.

In parasitic infections of the large intestine, we previously developed a model of intracecal infection by the protozoan *Entamoeba* *histolytica* in susceptible C3H/HeJ mice (a strain with a spontaneous mutation in the TLR 4 gene) to simulate the intestinal infection caused by this parasite in humans [138]. In this model, the oral therapeutic administration of bLf resolved the intestinal infection with amoebae at high efficiency, effect that was shown to be associated to an increased local expression of IL-4 and IL-6 and the elicitation of sIgA in cecum [103], as happened with the *Salmonella* infection. Both IL-4 and IL-6 are Th2 interleukins involved in the up-regulation of sIgA and IL-6, and also is a secondary inflammatory cytokine that exhibits pro- and anti-inflammatory properties [139,140]. Thus, oral bLf administration led to the anti-inflammatory response of mediators such as Th2 cytokines and IgA that collaborate in the protection against the parasite and maintenance of homeostasis with protective impact on tissue integrity.

On the other hand, in experimental assays conducted in mice infected with enterovirus, bLf treatment displayed protective action resulting from hampering the viral interaction with host cells [141]. However, in vitro assays indicated that bLf did not exhibit any suppressive activity against rotavirus [142]. In addition, assessment of rotavirus infection incidence in children, either treated or not with bLf, showed that bLf neither prevented the viral infection nor had an effect on the levels of IFN-γ or IL-10 as markers of pro- and anti-inflammatory interleukins, respectively [105]. Thus, therapeutic action of bLf by itself on viral enteritis is unclear; however, assays in suckling rats showed that administration of whey protein concentrate containing bLf as well as other bioactive components, favored the resolution of acute gastroenteritis caused by rotavirus infection [143]. This result suggests a synergic effect from the bulk rather than each single bioactive component that provided protection to enterovirus.

#### 3.1.3. Effect of Lactoferrin on Gut-Associated Sepsis

Sepsis is a life-threatening syndrome that results by the harmful effects of inflammatory response associated to gut systemic infections [140,144,145]. As in the case of infectious intestinal diseases, assessment of inflammatory parameters regulated by bLf in response to systemic infections have been analyzed in a limited number of infection models, induced by *E. coli* strains administered by enteral or parenteral routes [106,107,146]. In most cases, models mimic sepsis conditions found in human systemic infections by invasive enteropathogenic bacteria in immunocompetent hosts, or by microbiota members causing opportunistic nosocomial infections in neonatal or adult patients underwent solely parenteral nutrition, post-surgery antibiotic treatment, or immunosuppression, among other conditions [121,126].

Therapeutic action of bLf and the synthetic peptide LFchimera was tested in BALB/c mice infected by intragastric route with enterohemorrhagic *E. coli* (EHEC); the latter is a food borne pathogen causing mild diarrhea, hemorrhagic colitis and, in some patients, hemolytic uremic syndrome characterized by anemia, thrombocytopenia and kidney injury [106]. In infected mice that underwent both Lf and LFchimera treatments, fecal bacterial output and kidney colonization were found reduced, while LFchimera significantly decreased mortality rate. Histological analysis in hematoxylin–eosin stained tissue slices to assess the inflammatory response reveled that both Lf and LFchimera treatments decreased kidneys damage in comparison with mice without bLf treatments that showed tubular necrosis, glomerular injury and intratubular hyaline cast.

Furthermore, in vivo and in vitro assays have documented the role of r-hLf against gut-related systemic infection by *E. coli* strain Ec5 causing meningitis in orogastrically infected neonatal rats [146]. This model mimics clinical observations in neonates which evidence that parenteral feeding as sole route of nutrition is a risk factor of sepsis, resulting from translocation of microbiota members, e.g., Gram-negative enterobacteria, from intestinal tract to systemic organs via bloodstream, with potentially fatal consequences. Treatment with r-hLf decreased the clinical sepsis illness and bacterial loads in the kidney and blood while in vitro assays in macrophage cultures showed that levels of nitric oxide, TNFα and NF-κB expression elicited by LPS were even higher following the addition of r-hLf. These findings suggest that protective action of r-hLf resulted from an optimal activation of macrophages via pro-inflammatory cytokine elicitation to enhance their bacterial killing activity [146].

Effect of bLf against sepsis caused by *E. coli* and *Staphylococcus aureus* has been demonstrated in cyclophosphamide immunosuppressed mice, as a model to mimic immunosuppressive therapy for autoimmune and neoplastic diseases. Antisepsis action of bLf evidenced by the reduction of bacterial load in the spleen and liver was associated with a rise of circulating leukocytes induced by the elicitation of IL-6 in the spleen as well as peritoneal macrophages in immunosuppressed mice treated with bLf [108]. Studies in mice systemically infected with *E. coli* evidenced that bLf enhanced the killing activity and recruitment of neutrophils [107,147,148]. Action of bLf against *E. coli* sepsis resulted from the elicitation of IL-1 involved in the production of acute phase proteins, as well as from the amelioration on TNFα levels increased in response to the infection [107].

Since high levels of circulating pro-inflammatory cytokines such as TNFα have lethal consequence, the results described above suggest that the ability of bLf to control the TNFα increase may underlie its protective action to sepsis, as found in in vitro and in vivo assays [107,146]. Clinical trials have confirmed the potential application of bLf to prevent nosocomial sepsis and necrotizing enterocolitis of premature neonates, diseases that were associated with the up-modulation of T regulatory cells (FOXP3+ CD4+, and CD25hi) involved in the control of intestinal immune response against pathogens, strengthening the essential role of bLf in the control of the intestinal homeostasis [149].

#### 3.1.4. Gut and Systemic Lipopolysaccharide (LPS)-Related Inflammation

Pro- and anti-inflammatory properties of Lf in bacterial infections have been ascribed to its ability to act as a LPS-binding protein (LBP). Modulatory effects of either hLf or bLf, including derived Lfcins, on parameters associated with inflammation, has been evidenced in models of intestinal and systemic-related endotoxemia induced by LPS (also known as endotoxin) from Gram-negative enterobacteria [150,151]. These experimental models are intended to assess the impact of Lf at intestinal and systemic levels on inflammatory markers elicited by LPS that affect the gut barrier, bacterial translocation, diarrhea, tissue damage and endotoxic shock, among others [110,152,153,154,155]. In some cases, experimental assays with LPS administration encompass the use of zymosan, d-galactosamine (d-GalN) or carrageenan to increase the sensibility of animals toward the toxic effects of low LPS doses [153,154].

Porcine Lf (pLf) bioactive derivatives such as pLf peptide 20 (LFP20) have been proved to confer protection in mice against LPS damage to colon, associated with its down-modulatory action on pro-inflammatory cytokines TNFα, IL-6 and IFNγ. LFP20 elicited the expression of tight junction proteins involved in the regulation of intestinal permeability, i.e., zonula occludens-1, occludin and claudin-1, as well as decreased colonic apoptosis [110,152,153,154,155,156,157]. Protective action of hLf on the gut barrier function against the LPS-induced intestinal damage has also been described for hLf in in vitro cultures of Caco-2 cells and jejune segments of mice underwent LPS-endotoxemia [155,158]. Iron status of bLf had no impact on parameters of the gut barrier function, as documented in Caco-2 cell cultures incubated with murine (J774A.1) macrophages; however, apo-bLf displayed stronger neutralizing effects than holo-bLf against the pro-inflammatory cytokine generation in response to LPS and thermally inactivated *E. coli* CM226 antigens, suggesting that iron content may determine the protective role of Lf toward the inflammation caused by gut endotoxemia and/or sepsis [44].

At intestinal level, neutralizing activity of bLf or hLf has been tested in models of endotoxemia in mice induced by the intraperitoneal administration of LPS [152,155]. Other models of LPS neutralization by bLf include enterogenic endotoxemia in rats injected with carrageenan by intraperitoneal route, and also infection with *E. coli* in rats treated with nebacetin by intraduodenal via to enable the LPS release [154]. In the model of mice endotoxemia, intraperitoneal administration of hLf provided protection against deleterious effects of LPS on the intestinal integrity [155]. Moreover, bLf administered by intraperitoneal route in mice attenuated the LPS-induced diarrhea by decreasing the production of pro-inflammatory mediators with powerful diarrheagenic activity, i.e., prostaglandin E (PGE) by enterocytes in the small intestine and nitric oxide in plasma [152]. In the enterogenic endotoxemia model in rats, bLf decreased the endotoxic activity of LPS, as measured in plasma on a dose-dependent manner, and also decreased the bacterial loads in the mesenteric lymph nodes [154]. Some experimental assays in neonatal mice indicated that feeding with bLf and/or bifidobacteria decreased the intestinal levels of LPS without changes in cell populations producing TNFα, IFNγ and IL-6 in Peyer’s patches [159].

At the systemic level, the neutralizing activity of hLf-derived LF33 peptide has been tested in models of endotoxemia in mice, induced by intravenous administration of LPS and d-GalN. In this assays, LF33 exhibited protective activity against lethal intravenous LPS challenge by decreasing circulating levels of TNFα [110,146]. The dual action of Lf on circulating TNFα seems to underlie its protective role, since down-modulation of TNFα provided protection against the deleterious impact of endotoxemia, whereas the modulatory role of Lf in the control of TNFα elicitation is critical for eradication of gut-related systemic infections [108,110,146].

In another example, bLf displayed prophylactic action against lethal shock occurring upon intravenous injection of LPS in germ-free piglets; this is a valuable model to study the primary toxicity of endotoxin portion of LPS, i.e., lipid A, rather than the secondary toxicity of O and R polysaccharide portions [160]. In cultures of monocytes from LPS-treated piglets, bLf inhibited the interaction of LPS with CD14, an antigen surface marker expressed by mononuclear phagocytes. Priming of these phagocytic cells by LPS via CD14 ligation, resulted in the elicitation of powerful pro-inflammatory cytokines including TNFα, IL-1 and IL-6 with lethal outcome for host in endotoxemic conditions [161]. Moreover, oral administration of bLf prior to an intravenous LPS challenge in piglets provided protection against the mortality caused by LPS-induced hypothermia [160]. Like bLf, protective activity against hypothermia has been described with hLf in mice underwent LPS-endotoxemia [155].

Effect of bLf on LPS-neutralization and *E. coli* bacteremia has been explored in LPS toxicity susceptible C3H/HeCr mice carrying a defective *tlr-4* gene as well as in CBA mice resistant to LPS [162,163]. Unlike its protective activity in CBA mice, bLf failed to confer protection against endotoxemia and *E. coli* bacteremia in C3H/HeCr mice due to its inability to ameliorate the elicitation of TNF-α and IFNγ, and to prevent IL-6 decrease. Thus, an unbalanced cytokine response may be responsible of the high susceptibility to LPS endotoxemia in C3H/HeCr mice [163].

Differential impact of concurrent, prophylactic, and therapeutic effects of intraperitoneal administration of hLf on endotoxemia were analyzed in mice by intravenous administration of LPS [109]. In the concurrent scheme, hLf administered prior to the LPS challenge decreased the serum levels of TNFα, nitric oxide, IL-6 and IL-10. In the prophylactic and therapeutic protocols, hLf significantly down-modulated serum TNFα and nitric oxide, but no significant fluctuations were seen in the levels of IL-6 and IL-10.

In a mice model of hepatitis induced by intraperitoneal co-administration of LPS and zymosan, orally administered bLf decreased the serum aspartate aminotransferase activity (a marker of liver inflammation), and increased in the small intestine the production of IL-11, an anti-inflammatory cytokine with a role in the amelioration of inflammatory response [153,164]. The findings indicate that the up-modulation of IL-11 levels by bLf seems to provide therapeutic action in the small intestine induced by LPS-zymosan in this model of hepatitis. Treatment with r-hLf has also been analyzed in the same mice models of induced hepatitis [165]. In the experimental scenario, r-hLf provided protection against hepatitis development as determined by the decrease in alanine transaminase activity as the marker of liver damage, which was associated with down-modulation of serum TNFα levels. Moreover, the protective effect of r-hLf was not found in mice pre-treated with gadolinium chloride that destroys Kupffer cells, suggesting that these cells are the source of TNFα and the targets of r-hLf [165].

The mechanisms of Lf action on the inflammatory response have been analyzed in regarding to the ability of Lf to interact with LPS and block its interaction with TLR4 in in vitro cultures of intestinal cell lines and murine peritoneal or cell line macrophages [156,157,166,167,168,169,170]. These assays have evidenced that Lf down- or up-modulates LPS-mediated inflammatory response via dependent or independent TLR4/NF-κB signal pathway. Down-modulatory impact on LPS-mediated inflammation was found in assays of intestinal porcine epithelial cell line 1 (IPEC-1) treated with the pLf-derivative peptide LFP20, effect that resulted from its ability to inhibit MyD88/NF-κB and MyD88/MAPK signaling pathways [156,157]. Similarly, in human monocyte cell lines (THP-1 and Mono Mac 6), hLf also displayed down-modulatory activity on LPS-associated cytokine response by blocking the binding of NF-κB to the TNFα promoter [170]. Assays in cultures of human peripheral blood mononuclear cells showed that bLf displayed an anti-inflammatory response by driving the differentiation of LPS-treated monocytes toward dendritic cells with low capacity of both differentiation and elicitation of Th1 response by counteracting the TLR4 mediated activation signals [167]. On the other hand, up-modulatory impact of Lf on inflammatory effects of LPS was documented in assays of RAW 264.7 macrophage cell line and peritoneal macrophages, indicating that bLf, in the form of complex with LPS, enhanced cytokine response of TNFα, IL-1 and IL-6 via TLR4–NF-κB signal pathway [168]. However, another study suggests that the up-modulatory effect of Lf on LPS-mediated inflammation may involve pathways other than TLR4 signaling. Thus, assays on RAW 264.7 macrophage cell line treated with LPS showed that elicitation of IL-6 levels by bLf was TLR4-independent [169]. Therefore, Lf displayed a dual role on the LPS-mediated response of pro-inflammatory cytokines that encompasses alternative routes of TLR4 signalization. Other presumable mechanism of LPS-mediated inflammation includes the ability of Lf to control the expression of iron-regulating proteins as found in THP-1 monocyte/macrophage cultures; in these cells, Lf prevented the LPS-induced decreased of ferroportin by reducing the IL-6 levels [166]. As we mentioned before, ferroportin is an iron binding protein involved in the control of iron levels during inflammatory response.

In summary, the findings described above provide the experimental evidence that support the protective role of Lf against the deleterious effects of LPS-induced pro-inflammatory cytokine response on the gut-barrier function, diarrhea, bacterial translocation, and tissue damage. Having in mind the antimicrobial and LPS-binding protein activities of Lf, its application either alone or in combination with probiotics, or as an adjunctive compound of antibiotics, may represent a very promising strategy for the treatment and prevention of sepsis and endotoxic shock.

### 3.2. Respiratory Tract

#### 3.2.1. Respiratory Tract Inflammation and Infections: An Overview

The human respiratory tract (RT) is responsible for the mobilization of millions of liters of gases throughout life. Delivery of life-requiring oxygen to the systemic circulation and organs implies the potential incorporation of countless particles, toxicants and microbes, which are countered by local innate and adaptive immune responses that avoid their entry into the lung tissue and circulation and protect the lung structure and function [171]. Infections in the RT are very frequent in the population and represent a considerable cause of worldwide morbidity and mortality [172]. Infections of the upper RT such as common cold, laryngitis, pharyngitis, epiglottitis, otitis and sinusitis are typically caused by virus, bacteria, and, at less extension, by fungi. As an example, the common cold is a viral disease considered as the most frequent infection in humans, which can be caused by rhinoviruses, coronavirus, parainfluenza and adenovirus, and less frequently by respiratory syncytial virus and enterovirus. However, the influenza virus, a common cause of seasonal flu, can simultaneously affect other parts of the RT, including the lower tract. Infections of the lower RT are mainly caused by bacteria, but also by virus, fungi and even parasites. They include bronchitis, pneumonia and pulmonary abscesses, among others. Tuberculosis, caused by the bacterium *Mycobacterium tuberculosis*, is among the most prevalent infections in the lower RT, causing mainly pneumonia, but also affecting other organs [172].

#### 3.2.2. Modulatory Effects of Lactoferrin on the Infection-Associated Inflammatory Response in the Respiratory Tract

All RT infections are accompanied with an inflammatory process whose intensity depends on many variables, including the type of pathogen and its virulence, the inoculum, the affected tissue, the host immunological status and whether the infection is acute or chronic. Although the inflammatory process is essential for the control of invasive infectious agents, the development of an exacerbated or chronic inflammation results in alterations of the respiratory capacity due to the lung tissue damage, including edema, increased airway resistance and mucus production, such as in the infection with influenza virus [173]. Understanding how inflammation alters the respiratory system is indispensable for the development of better therapeutic interventions to support breathing and lung plasticity as a clinical treatment. In this regard, evidence of the modulation of RT inflammation by bLf associated to certain microbes has been reported using several in vitro and in vivo models. Moreover, Lf is considered the second most important antimicrobial and anti-inflammatory peptide after lysozyme in the upper RT [174]. Tuberculosis is by far the most studied in vivo model of microbial pulmonary infection. This bacterial infection affecting nearly a third of the world’s population and having a rate for new infections of approximately 0.6% per year [175], is targeted by the Bacillus Calmette-Guerin (BCG) vaccine, the most widely used vaccine in the world which has remained almost unchanged since 1921 [176]. A study carried out in 2005 showed that a single subcutaneous immunization of mice with a mix of BCG and bLf emulsified with Freund’s adjuvant, followed by a challenge with *M. tuberculosis* in aerosol, resulted in decreased mycobacterial loads in the lungs and spleen [111]. Splenocyte proliferative response to heat-killed BCG showed increased IL-12 and IFNγ production. In a subsequent study by the same group, it was showed that bLf admixed to the BCG vaccine, in incomplete Freund’s adjuvant or PBS, increased mice protection against a *M. tuberculosis* challenge when compared with mice that received BCG alone [112]. In addition, there was a significant reduction of lung bacterial load associated to increased production of IFNγ and IL-6 by splenocytes, as mentioned before; in this study, bLf addition to the vaccine also resulted in a clear reduction in lung pathology, concomitant with down-regulation of pro-inflammatory mediators TNFα or IL-1β, suggesting that the main action of Lf to enhance the BCG vaccine relied on its immunomodulatory properties reducing the immune-related tissue pathology, in part, by modulating macrophages and dendritic cells ability to present antigens and stimulate T-cells [177]. Noteworthy, the lymphocytic recall response towards BCG antigens two months after infection was higher in the mice that received Lf as adjuvant, suggesting that Lf improved the specific T-cell Th1 response as determined by the increase in INFγ production [112].

The potential of r-hLf to reduce the *M. tuberculosis* tissue damage and pulmonary histopathology was also demonstrated in ulterior studies of the same group. They showed that r-hLf produced in the yeast *Pichia pastoris* expression system with a glycosylation pattern similar to its natural human neutrophil counterpart, in contrast to the non-glycosylated r-hLf, was able to improve the efficacy of the BCG vaccine in protecting against the challenge with *M. tuberculosis* in aerosol, as manifested primarily in a significant reduction in the associated pulmonary pathology [178]. In this case, the mycobacterial loads in the lung and spleen were not significantly reduced in the BCG-r-hLf group compared with the controls treated with BCG alone, but rather, the protection was associated with changes in the pathological manifestation of the lung disease; this was probably due to the notable immunomodulatory function of the granulocytic Lf used in this study, when compared with the Lf form from secretions, which is more microbicidal [178]. Recently, a r-hLf expressed in Chinese hamster ovary (CHO) cells was also used in the mixture with the BCG vaccine, showing a slight decrease over the time in the lung pathology after aerosol challenge with *M. tuberculosis*, which correlated with an initial increase in the secretion of inflammatory cytokines followed by their posterior decrease [113].

The efficacy of bLf in enhancing the BCG vaccine action was more recently analyzed using a more amenable route of administration. In this study, mice that received drinking water containing 0.5% bLf at Day 0 or 7 post-infection had lower Colony Forming Units (CFU) and lower inflammation in the lungs, with increased numbers of IFNγ producing T CD4 and CD8 cells and IL-17 producing lymphocytes when compared with animals vaccinated with BCG alone [114]. Noteworthy, bLf did not affect the in vitro replication of *M. tuberculosis* but instead enhanced the killing of bacteria by macrophages in a nitric oxide dependent way [114]. These studies using the models of pulmonary infection with *M. tuberculosis* suggest that Lf promotes certain up-regulation of pro-inflammatory response, while down-regulating overall tissue immunopathology.

The role of Lf in the inflammation in other pulmonary infections has been less addressed. Homeostatic effect of bLf on inflammation was reported in in vitro cultures of cystic fibrosis (CF) bronchial cells (IB3-1) infected with *Burkholderia cenocepacia*, a Gram-negative opportunistic bacterium that recurrently infects patients with CF forming biofilms and is usually highly resistant to currently available broad-spectrum antibiotics. Thus, even though the addition of bLf did not reduce the rates of bacterial invasion, it decreased the release of pro-inflammatory IL-1β, while augmented the secretion of anti-inflammatory IL-11, suggesting a role for bLf in protecting CF bronchial infected cells from the inflammation-associated damage [115]. Noteworthy, this study correlated with a previous one addressed on sputum samples from patients with CF which showed an inverse association between the levels of Lf in the secretions and the inflammation burden [179]. Another study showed that the decrease of Lf levels in patients with CF was due to its cleavage by the increased cathepsin activity in *Pseudomonas aeruginosa*-positive sputum samples, another biofilm-forming opportunistic pathogen of these patients. A similar result with Lf and Tf undergoing proteolysis was previously reported in bronchioalveolar lavage of *P. aeruginosa* infected CF patients [180]. These results suggest that the proteolytic cleavage of Lf in patients with CF can contribute to *B. cenocepacia* and *P. aeruginosa*-associated lung damage, and that infection-associated lung damage can be improved by the exogenous therapeutic administration of Lf, due to its potent immunomodulatory properties.

Similar protective effect of bLf has been found in a murine model of lung injury induced by intraperitoneally administered LPS [116]. In this study, the intraperitoneal injection of bLf (5 mg/mouse) 1 h before (prophylactic effect) or 1 h after (therapeutic effect) LPS challenge, were associated with significant reduction of the total number of leukocytes in bronchioalveolar lavage samples, increased IL-10, and decreased TNFα concentrations and myeloperoxidase activity [116]. These changes paralleled attenuation of lung edema and inflammatory infiltration, suggesting a protective role of bLf by avoiding the damage caused by the LPS-induced acute inflammatory response.

However, the protective role of Lf based on the regulation of inflammation is not observed in all cases of RT infections. There are several viral infection models where the immunomodulatory effect of Lf has not been documented. Thus, although Lf has in vitro antiviral activity against the Respiratory Syncytial Virus (RSV) [181], as well as an immunomodulatory effect reducing the release of IL-8 by Hep-2 cells infected with RSV [182], the oral or intraperitoneal administration of different doses (2 to 10 mg/animal/day) of bLF to mice from 48 h before until 96 h post-RSV infection did not have any effect on viral loads, pulmonary airflow resistance or obstruction, degree or type of pulmonary inflammation and serum T cellular responses, evaluated on Day 5 post-RSV infection [117]. Similar result was observed in a study carried out in mice treated by intranasal route with bLf on Days 2–5, and evaluated on Day 6 post-RSV infection [118]. Another example is a mouse model of influenza infection, where the daily oral administration of 62.5 mg of bLf from 24 h before infection, did not have any effect on viral load and concentration of IFNγ, IL-6 and IL-12 cytokines evaluated at six days post-infection, when compared with untreated infected mice [119]. The reason for the lack of bLf protecting effect in these viral infection mice models is unknown, but possible explanations could be related to timing, dosing, and route of bLf administration.

In contrast, oral bLf and curcumin supplementation to children with recurrent viral RT infections resulted in immune modulation by modifying the lymphocyte population and cytokine responses that reduce the rate of infections [183]. A recent study aimed to determine the effect of three months supplementation with bLf-fortified formula on respiratory tract infections and diarrhea in 260 Chinese weaned infants (4–6 months age), showed similar results, with a reduction in the incidence rate of respiratory-related illnesses when compared with a placebo group [184]. Moreover, a study in patients with chronic rhinosinusitis has established an association between the genetic deficiency of Lf synthesis in the upper RT and the increased susceptibility of certain individuals to bacterial colonization, biofilm development, and recalcitrant sinus disease [185]. This new knowledge of Lf immunomodulation paves the way to more general design of T cell-dependent vaccines that incorporate naturally occurring granulocytic components, which may be useful in infectious diseases to reduce immune-mediated tissue damage.

### 3.3. Modulatory Effects of Lactoferrin on Other Infection-Associated Inflammatory Processes Inflammation

#### Colostrum, Milk and Mastitis by Staphylococci

The role of bLf on inflammatory response associated to infection has been described in other mucosal sites, such as the mammary gland of cows suffering staphylococcal mastitis [120]. Assessment of mammary gland secretions showed that, in sick cows, intramammary infusion of bLf decreased the numbers of staphylococci and increased C3 levels, whereas in healthy animals bLf infusion increased the numbers of PMN leukocytes expressing CD11b, an integrin when complexed with CD18 (CR3) acts as receptor for the iC3b complement fragment [120]. According to these findings, up-modulatory effect of bLf on pro-inflammatory components of innate immunity may underlie its therapeutic action toward mastitis; in fact, alternative approaches have also been tested to decrease the deleterious effect of inflammation on tissue integrity. Combination of bLf and antibiotics was found to be effective to control the staphylococcal mastitis and to attenuate the mRNA expression of TNFα via the inhibition of NF-κB activation [121]. Thus, this approach may contribute to decrease the effects of inflammation on tissue damage and also to reduce the antibiotic dosage for the eradication of staphylococcal infection by multiresistant strains. Inflammatory response in staphylococcal mastitis seems to be correlated with the elicitation of peptides derived from bLf-elastase proteolysis that display low concanavalin A and low iron-binding affinities, as well as antibacterial properties, but induce the expression of pro-inflammatory cytokines IL-8 and TNFα leading to neutrophil infiltration [122,123]. Like staphylococcal mastitis, elicitation of low ConA affinity Lf-peptide derivatives in parotid saliva was correlated with the severity of symptoms of periodontitis patients [186].

In oral cavity, the modulatory action of bLf on infection-associated inflammatory response has been documented in mice infected with *Candida albicans* [187]. Experimental settings of oral candidiasis in immunosuppressed mice showed that oral administration of bLf displayed therapeutic effect by inhibiting the suppressive effects of infection on inflammatory parameters of innate response, such as circulating PMN neutrophils and cervical lymph-node cells; moreover, generation of IFNγ and TNFα was found increased in cervical lymph-nodes cultures primed with heat-killed *C. albicans* from bLf-treated mice [124]. According to in vitro assays testing full length bLf and bLf-derived peptides, therapeutic effect of parental bLf relies on the N-terminal portion associated with its ability to up-modulate the killing action of PMN neutrophils by increasing the superoxide generation, protein kinase C, p38 MAPK activity, and the expression of p47phox [187]. These findings suggest that, in the murine model of candidiasis, the up-modulatory effect of bLf on parameters of inflammation provides protection to *C. albicans* infection.

Data from in vivo and in vitro assays support the role of bLf and hLf in up- and down-modulation of inflammatory response to extra-intestinal infections, such as hepatic amoebiasis [62], listeriosis [125,188], urinary tract infections [126], legionellosis [189], and staphylococcal septicemia [190]. In experimental amoebic liver abscess in hamsters, bLf treatment by gavage had protective action against the hepatic lesions by *E. histolytica* and favored the normalization of the liver function [62]. Having in mind its intrinsic anti-inflammatory and therapeutic action, as well as its low toxicity, the use of bLf as adjunct of conventional drugs such as metronidazole, may contribute to decrease the drug dosage in the treatment of amoebiasis and even decrease the drug resistance of the parasite.

In vitro experiments on *Listeria monocytogenes* infection in IFNγ primed THP cell cultures indicated that bLf displayed a protective action against cell death by necrosis, whereas bovine Lfcin B diverted the death cell from necrosis to apoptosis [188]. Findings indicated a protective role of bLf and Lfcin B by reducing the inflammatory response associated to necrosis caused by the intracellular infection of the pathogenic bacteria in macrophages, and favored the anti-inflammatory conditions of cell death by apoptosis on these cells. Data from studies of infection with *L. monocytogenes* in mice indicate that treatment with hLf displayed significant effects on one main organ target of this pathogen in regards to bacterial colonization, necrosis, and mRNA expression of pro-inflammatory cytokines TNFα, IL-1β and IFNγ [125].

Although the majority of the experimental data on Lf properties has been obtained with bLf, some experimental studies indicate that hLf displays even more potent antibacterial and anti-inflammatory action. Murine model of infection of urinary bladder with the uropathogenic *E. coli* O6K5 strain showed that perorally administered hLf decreased the bacterial load in the kidneys and urinary bladder as well as the inflammatory response, as evidenced by reduced IL-6 levels in urine at 2 h post-infection, and in plasma, at 24 h post-infection [126]. Thus, hLf administered by peroral route provides therapeutic action against infection and inflammation in remote sites such as the urinary tract.

In vitro assays of *Legionella* *pneumophila* infection in cultures of monocytes from healthy volunteers indicated that unlike holo-hLf that promoted the bacterial growing, apo-hLf inhibited the intracellular multiplication of the pathogen in both inactivated and IFNγ activated monocytes [189]. These findings suggest a synergy between the antibacterial effect of iron-free human lactoferrin and the stimulating killer effects of the pro-inflammatory cytokine IFNγ on infected cells, leading to conditions for control of bacterial multiplication inside cells. A model of *S.* *aureus* infection in hLf-transgenic mice showed the pivotal role of hLf in the elicitation of a Th1 profile of cytokines, which determined the resolution of systemic infection. Polarization of the immune response to the Th1 profile in hLf-transgenic mice was evidenced by up-modulation of TNFα and IFNγ levels and down-modulation of Th2 cytokines IL-5 and IL-10 in culture supernatants of spleen cells [127].

In summary, the studies mentioned above show that Lf and its derivative peptides display bimodal effects that provide conditions for the up- and down-regulation of inflammation leading to the resolution of extra-intestinal infections. The biotechnological development of formulations of Lf as nanoparticles for potential clinical use, in addition to Lf-hydrolysate, and native Lf alone or in combination with probiotics, may have application for the control of infections and inflammation [6,74,130,184,190,191].

## 4. Perspectives

Evidence from the basic studies in animals of experimentation about the prophylactic and therapeutic activity of Lf as antimicrobial and modulatory agent on inflammatory response, have promoted this glycoprotein from the innate immune system as a focus of interest for the biotechnological development of nanoparticle-based formulations for potential clinical use. In addition, Lf-hydrolysate, and native Lf alone or in combination with antibiotics and probiotics, may have potential application in the control of neonatal infections, and in inflammation. More studies are necessary to support the generalized practical application of Lf, mainly in the control of inflammation associated to infections.

## Figures and Tables

**Table 1:** Modulatory effects of lactoferrin on inflammation associated to infections.

Model	Finding	Reference
	Gastrointestinal infections	
Bovine Lf (bLf) treatment of rabbits infected with *Shigella flexneri* (*S. flexneri)*	↓Gut inflammation (microscopic observation), ↓submucosal edema, ↓infiltration of leukocytes	[98]
bLf treatment of BALB/c mice infected with *Salmonella enterica* (*S. enterica*) serovar Typhimurium	↓Gut inflammation, ↓necrosis	[99]
bLf treatment of Caco-2 cell-line cultures infected with *Escherichia coli* (*E. coli*) HB101 (non-invasive), or recombinant *E. coli* HB101 pRI203 (invasive)	↓IL-8 mRNA expression in Caco-2 cells infected with *E. coli* HB101; ↓IL-6, ↓IL-8, ↓Tumor Necrosis Factor (TNF)α levels in Caco-2 cells infected with *E. coli* HB101pRI203	[100]
bLf treatment of cultured cells infected with *E. coli* LF82 and biopsies from patients with Crohn’s disease	↓IL-6, ↓IL-8 and ↓TNFα mRNA expression	[101]
bLf treatment of intestinal cell cultures infected with *E. coli* LF82 isolated from Crohn’s disease patients	↑Ferroportin (Fpn) in infected cells suggesting that bLf action on inflammatory response in epithelial cells involves the iron homeostasis	[102]
bLF-nanoparticles (bLf-nano) administration to BALB/c mice infected with *S. enterica* serovar Typhimurium	↑TNF1α, ↑Interferon (IFN)1β and ↑IFNIII levels (proinflammatory cytokines)	[6]
bLf administration to C3H/HeJ mice infected with *Entamoeba histolytica* (*E. histolytica*)	↑IL-4 (Th2), ↑IL-6, ↑IgA ↓damage and ↓Inflammation	[103]
bLf treatment to BALB/c mice infected with *Helicobacter pylori* (*H. pylori)*	↓Gastric colonization and ↓inflammation (histopathology score)	[104]
bLf treatment of rotavirus infection children	↔IFNγ, ↔IL-10 and ↔rotavirus incidence in children whether fed or unfed with bLf	[105]
	Gut-related systemic infections (sepsis)	
Administration of bLf or synthetic LF chimera peptide to BALB/c mice infected with enterohaemorrhagic *E. coli* (EHEC) 0157:H7	↓Mortality (only LF-chimera); ↓kidney damage; ↓fecal bacterial output and ↓sepsis: bLf and LFchimera	[106]
	Septicemia	
Single-dose bLf administration 2 or 24 h prior intravenous infection of CBA mice with *E. coli* O55 strain	↓Blood TNF1α (24 h), or ↑blood TNFα (2 h)	[107]
Lf administrated with drinking water (0.5% solution) during 14 days in mice immunosuppressed by cyclophosphamide treatment, and intravenously infected with *E. coli*	↓*E. coli* bacterial load in spleen and liver, ↑blood neutrophils, ↑IL-6 in spleen and peritoneal cells	[108]
	Endotoxemia	
Prophylactic or therapeutic effect of bLf on systemic inflammation in mice treated with lipopolysaccharide (LPS)	↓TNFα, ↓IL-6 and ↓IL-10, 1 h prior to the LPS treatment (prophylactic effect). ↓TNFα, ↓Nitric Oxide (NO), ↔IL-6, ↔IL-10, 18 h prior to the LPS treatment (prophylactic effect). ↓NO and ↓post-shock, 2 h after LPS treatment (therapeutic effect)	[109]
LF33 peptide administration to mice treated with LPS and to RAW 264.7 cell-line cultures treated with LPS	↓Limulus amoebocyte lysate coagulation, ↓TNFα secretion by RAW 264.7 cells induced by LPS, ↓TNF-α levels correlated with protection to lethal LPS-induced septic shock	[110]
	Respiratory tract infections	
Adjuvant effect of Lf mixed with bacillus Calmette-Guerin (BCG) vaccine on mice infected with *Mycobacterium tuberculosis* (*M. tuberculosis)*	↓Lung infection, ↑IFNγ, ↑IL-12 in spleen cell cultures, ↓TNFα and ↓IL-1β correlated with ↓lung pathology. ↑lymphocytic recall response towards BCG	[111,112]
Recombinant human Lf mixed with BCG vaccine in mice infected with *M. tuberculosis*	Early↑ and late↓ of pro-inflammatory cytokines that correlated with the ↓lung pathology	[113]
bLf effect in enhancing BCG vaccine by oral route in mice infected with *M. tuberculosis*	↓Colony Forming Units (CFU) and ↓inflammation in the lungs, ↑IFNγ producing T CD4 and CD8 cells and ↑Il-17 lymphocytes	[114]
bLf effects on cystic fibrosis and bronchial IB3-1 cell cultures infected with *Burkholderia cenocepacia* (*B. cenocepacia*)	↓IL-1β (pro-inflammatory cytokine), ↓IL-11 (anti-inflammatory cytokine)	[115]
bLf administration to a murine model of lung injury by LPS	↓Bronchioalveolar leukocytes, ↓TNF-α, ↓myeloperoxidase (MPO) activity, ↑IL-10, ↓lung edema and inflammation	[116]
bLf administration to a murine model of respiratory syncytial virus infection	↔Viral loads and ↔lung inflammation	[117,118]
bLf administration to a murine model of influenza	↔Viral load and ↔IFNγ, IL-6 and IL-12 in the lungs	[119]
	Other mucosal and systemic sites	
bLf effects on mammary gland in cows with *Staphylococcous aureus* (*S. aureus*) mastitis	↓Bacterial load, ↑C3 levels, ↓TNFα mRNA expression via Nuclear Factor κB (NFκB) inhibition, ↑curation, ↑proinflammatory cytokines is correlated with ↑peptides derived from bLf-elastase proteolysis	[120,121,122,123]
bLf effects on oral candidiasis in immunosuppressed mice infected with *Candida albicans* (*C. albicans*)	bLf blocked the suppressive effects of candidiasis in Polymorphonuclear (PMN) neutrophils; ↑IFNγ and TNFα production in cervical lymph nodes	[124]
bLf effects on hamsters with amoebic liver abscess by *E. histolytica*	No damage or inflammation in the liver	[62]
Human Lf (hLf) effects on BALB/c mice infected with *Listeria monocytogenes* (*L. monocytogenes*)	↓Bacterial load and ↓necrotic foci in the liver, ↔necrotic foci in the spleen, ↓TNFα, IL-1β and IFNγ mRNA	[125]
hLf and peptide-hLf derivatives administration to C3H/TiF mice infected with *E. coli* O6K5 uropathogenic strain	↓Bacterial load in the bladder and kidneys, ↓leukocyte in urine, ↓urinary IL-6 levels at 2 h and systemic IL-6 levels at 24 h post-infection	[126]
hLf expressing transgenic mice infected with *S. aureus*	↓Bacterial growth, ↓septicemia, ↓mortality than congenic litter mates. ↑Th1 polarization in the spleen, given that: ↑TNFα and ↑IFNγ, ↓IL-5 and ↓IL-10 upon stimulation ex vivo with exotoxin toxic shock syndrome toxin-1 compared with congenic controls	[127]

↓ decrease; ↑ increase; ↔ no changes.

## Abbreviations

apo-Lf	apo-lactoferrin (iron-free lactoferrin)
bLf	bovine lactoferrin
Lfcin B	bovine lactoferricin B
d-GalN	d-galactosamine
IFNγ	γ interferon
holo-Lf	holo-lactoferrin (iron-loaded lactoferrin)
holo-Tf	holo-transferrin (iron-loaded transferrin)
hLf	human Lf
IBD	inflammatory bowel disease
IL	interleukin
Lfcin	lactoferricin
Lf	lactoferrin
LfR	lactoferrin receptor
NF-κB	nuclear factor κB
ovoTf	ovotransferrin
PMN	polymorphonuclear
r-hLf	recombinant human lactoferrin
RSV	respiratory syncytial virus
RT	respiratory tract
sIgA	secretory immunoglobulin A
Tf	transferrin
TNFα	tumor necrosis factor α
